# The Properties of Damaged Starch Granules: The Relationship Between Granule Structure and Water–Starch Polymer Interactions

**DOI:** 10.3390/foods14010021

**Published:** 2024-12-25

**Authors:** Andrés Gustavo Teobaldi, Esteban Josué Carrillo Parra, Gabriela Noel Barrera, Pablo Daniel Ribotta

**Affiliations:** 1 Instituto de Ciencia y Tecnología de los Alimentos Córdoba (ICYTAC-CONICET), Universidad Nacional de Córdoba, Av. Filloy S/N, Ciudad Universitaria, Córdoba CP 5000, Argentina; ateobaldi@agro.unc.edu.ar (A.G.T.); ejocarrillo@mi.unc.edu.ar (E.J.C.P.); gbarrera@agro.unc.edu.ar (G.N.B.); 2 Departamento de Química Industrial y Aplicada, Facultad de Ciencias Exactas, Físicas y Naturales (FCEFyN), Universidad Nacional de Córdoba (UNC), Av. Vélez Sarsfield 1611, Córdoba CP 5000, Argentina

**Keywords:** starch, mechanical damage, granule–water interactions, granule structure, crystallinity

## Abstract

The morphology of wheat starch granules with different damaged starch (DS) content was analyzed using a particle size analyzer and scanning electron microscopy (SEM); the granular structure was studied using FT-IR spectroscopy and X-ray diffraction (XRD); and the granule–water interaction was evaluated by thermogravimetric analysis (TGA) and dynamic vapor sorption (DVS). The increase in the level of DS shifted the population of B-type granules towards larger particle diameters and shifted the population of A-type granules towards smaller particle diameters. The appearance of the surface of the starch-damaged granules was rough and flaky (SEM images). Crystallinity reductions were related to higher mechanical damage levels of the granular structure (FT-IR and XRD). Higher DS increased the liquid-water absorption capacity of the granules. Higher DS was associated with increments in less-bound water proportions and reductions in more strongly bound water proportions and related to reductions in the evaporation temperature of these water populations (TGA analyses). Concerning DVS data, the results suggested that the driving force for water–monolayer attachment to the starch granules decreased as DS increased. Therefore, it was suggested that the changes in granule structure led to a weaker water–starch polymer chain interactions due to the increase in DS. The results contribute to a better understanding of the influence of mechanical damage on the starch granular structure, which could be related to the rheological and thermal behavior of starch-based systems with different DS.

## 1. Introduction

Native wheat starch (NS) is a polymer macromolecule that consists primarily of linear amylose and highly branched amylopectin [1]. The polymer chains that constitute starch are organized into structures called granules. Native starch granules’ crystallinity varies from 15% to 45%. The semicrystalline solid characteristic of starch granules is determined by the structural arrangement of amylose and amylopectin. These polymers are arranged with their longitudinal axes perpendicular to the surface of the granule, giving rise to the structure in the form of concentric rings or layers typical of starch granules. Two types of layers can be identified, crystalline and amorphous, which are arranged alternately. The crystalline layers are mainly composed of amylopectin, specifically by the double helices of the amylopectin side chains, and in this matrix, some amylose molecules are dispersed. The amorphous layers originate at the branching points of amylopectin and are made up especially of amylose, which are arranged between the branches of amylopectin forming double helices. For most starches, the external surface of starch granules is the first barrier to processes such as granule hydration, enzyme hydrolysis, and chemical reaction with modifying agents. Consequently, it is becoming recognized that the nature of the granule surface, particularly the presence of surface proteins and lipids, may significantly affect the properties of the starch (Pérez et al., 2009) [2].

The process of grinding wheat until obtaining the desired particle size is achieved through applying compression, abrasion, shear and impact forces, and a combination of these. The granules that are damaged during milling constitute what is called damaged starch (DS). The degree of starch damage during milling is influenced by three main factors: the composition (amylose/amylopectin ratio) of the starch, the hardness of the grain, and the milling conditions. In hard wheat, damaged starch can constitute 8% or more of the total starch in the flour.

Damaged starch granules exhibit partial loss of birefringence, higher water absorption, and higher susceptibility to enzymatic hydrolysis. Damaged granules absorb between 200 and 430% of their weight in water at room temperature, while native granules absorb between 39 and 87%, increasing the flour’s water absorption capacity [3,4]. The polymer chains in the crystalline regions of the granules interact strongly among them, affecting the water penetration and allowing only water access to the amorphous regions. After the partial interruption of the crystalline order due to mechanical damage, water access is less restricted to these regions of the granule, increasing the water absorption capacity [5]. This condition makes damaged starch granules more accessible to amylases hydrolysis, compared to native ones, which results in the production of dextrins [6,7]. The degradation of starch molecules by milling occurs mainly at the α-(1→4) glycosidic bonds in the crystalline double helix, although it can also occur at the α-(1→6) branch points in the amorphous lamellae of starch granules. The breakdown of α-(1→4) glycosidic bonds in the crystalline region is denoted by the decrease in starch crystallinity after milling [8]. Molecules that form double helices in amorphous conformation are more flexible than crystalline ones, which helps reduce the impact of mechanical shear during milling [9,10]. Several authors [11,12] have shown that milling produces an alteration in the structure and functionality of starch granules. The damaged starch content influences the physicochemical and rheological properties of the flours, directly affecting the industrial performance of the flours [7,13,14]. Consequently, the damaged starch content of flour is of crucial importance in its characterization.

The objective of this work was to evaluate the influence of the milling damage level of wheat-starch granules’ structure on their hydration and water-holding properties, focusing on the mechanical process and its impact on the interactions between granules and water in liquid and gaseous states.

## 2. Materials and Methods

### 2.1. Samples

Native wheat starch (NS: 4.8% DS) was milled in a Whisper Series Bench Top disk mill (Rocklabs, Auckland, New Zeeland) at different times to obtain samples with different damaged starch levels. The temperature was controlled during the milling; it was kept under 40 °C to avoid starch oxidation reactions. Three levels of damaged starch were obtained after milling: DS1 (1 min in disk mill, 14.7% DS); DS2 (2 min in disk mill, 21.4% DS); and DS4 (4 min in disk mill, 32.2% DS). The damaged starch content was determined using the AACC 76–30 A method [15]. Each measurement was made in triplicate.

### 2.2. Particle Size Distribution Analysis

The particle size distributions of the samples were evaluated using a Laser Scattering Particle Size Distribution Analyzer (HORIBA Scientific, LA-960, Kyoto, Japan). The starch samples were placed in the dispersion tank of the particle size analyzer, which contained distilled water. For the measurements, a starch refractive index of 1.33 was selected. The analysis of particle size distributions was performed using LA-950 Software, which generated a multimodal report. All the measurements were carried out in duplicate.

### 2.3. Fourier Transform Infrared Spectroscopy (FT-IR)

Starch samples were analyzed using an infrared spectrometer. FT-IR spectra between 650 and 4000 cm^−1^ were obtained with a Nicolet iN10 infrared microscope with a cooled detector (Thermo Scientific, Waltham, MA, USA) and a resolution of 8 cm^−1^. The spectra obtained were analyzed with OMNICTM 8.3 software (Thermo Scientific, Waltham, MA, USA).

### 2.4. X-Ray Diffraction (XRD)

The degree of crystallinity of the samples was determined using X-ray diffraction with a diffractometer (Philips PW1800, San Jose, CA, USA). The X-ray patterns were obtained using Cu Kα radiation (λ = 0.154 nm) under the following operating conditions: 30 mA, 40 kV, a scanning range of 5–40° (2θ), and a scanning speed of 0.01 °/s. Both the crystalline and amorphous fractions were quantified, and the percentage of crystallinity was calculated following the procedure outlined by Barrera et al. [8]. Peakfit v4.12 software (Peakfit, Jandel Scientific, San Rafael, CA, USA) was used to deconvolute the diffractograms in order to quantify the crystalline peaks and amorphous phases.

### 2.5. Scanning Electron Microscopy (SEM)

Scanning electron microscopy (SEM) was used to evaluate the surface characteristics of the starch granules. The samples were mounted on a sample holder and coated with a 30 nm thick layer of gold using a sputter coating system. To carry out the observations, a Supra 55 VP scanning electron microscope (Carl Zeiss Co., Oberkochen, Germany) was used at an acceleration potential of 1 Kv. The observations were conducted using a secondary electron detector “SE” and an “In Lens”. Photographs were taken using automatic image capture software. Images were obtained with magnifications of 7000×–48,000×.

### 2.6. Water Absorption Capacity (WAC)

The water retention capacity (WAC) of the samples was determined using a method proposed by Palavecino et al. [16]. For the test, 0.5 g of sample (W1) was weighed in centrifuge tubes and 6 mL of distilled water was added. Each tube was incubated at 25 °C for 30 min and shacked at 0, 10, 20, and 30 min. The tubes were centrifuged for 20 min at 3000× *g* before draining the water excess. Each tube with wet sample (W2) was weighed and WAC was calculated as the coefficient between W1 and W2. Each measurement was made in duplicate.

(1) $$WAC=\frac{W2}{W1}$$

### 2.7. Thermogravimetric Analysis (TGA)

The thermogravimetric analysis (TGA) was carried out to analyze the interaction between water and starch granules. To standardize the moisture content of the samples, 2 g of the solid was placed in an airtight container along with a 15% *v*/*v* sulfuric acid solution. The containers were stored in a temperature-controlled chamber at 25 °C for 14 days until the equilibrium between ambient humidity and each sample was achieved. Then, hydrated starches (∼5 mg) were weighed into open aluminum capsules and analyzed over a temperature range of 25–120 °C at a rate of 4 °C/min. The temperature range used was selected (25–150 °C) to avoid starch decomposition. The graphs of the weight loss (considered as water loss) vs. temperature of each sample were obtained, as was the first derivative of these curves, that is, the rate of water loss (Derivative Thermo-Gravimetry (DTGA) (%/°C)). These curves were analyzed using TRIOS 5.1 software (TA Instruments, New Castle, DE, USA). The DTGA curves were analyzed by deconvolution to separate the overlapping peaks corresponding to different water populations through Gaussian functions and quantification areas (Peakfit 4.12, Jandel Scientific, San Rafael, CA, USA) [17]. The size of the peaks and the average temperature at which they occurred were determined. The size of the different peaks was calculated as the peak area (%) relative to the total peaks. Adjustments with R^2^ greater than 0.99 were considered. All the measurements were performed at least in duplicate.

### 2.8. Dynamic Vapor Sorption (DVS)

Dynamic vapor adsorption equipment, Advantage I model (Surface Measurement Systems Ltd., London, UK) was used. Approximately 25 mg of starch samples were weighed into the DVS pans and placed inside a chamber at a constant temperature (25 °C) and relative humidity (0% to 90%) controlled by the nitrogen flow. The equilibration moisture content at each humidity value was determined by a change in dm/dt of 0.002 (%/min). The determination of the adsorption and desorption curves was carried out in the range of 0 to 0.9 of P/P0 (partial pressure of water vapor inside the chamber/saturation pressure of pure water vapor at the same temperature). The pressure relationship that determines the aqueous activity of the gas inside the chamber was equal to the definition of the water activity (aw) of food at equilibrium [18]. Measurements were carried out in duplicate.

The theoretical models of Guggenheim, Anderson, and de Boer (GAB) and Brunauer–Emmett–Teller (BET) were adjusted to the experimental data (regression coefficient: 0.99) using SigmaPlot 10.0 software (Systat Software, Inc., Surrey, Germany). Nonlinear regression analysis was applied over the range 0–0.9 aw in the GAB model and 0–0.5 aw in the BET model to determine the apparent monolayer coverage. In those models, W was the equilibrium moisture content (g H_2_O/100 g dry solids), Wm was the monolayer content (g H_2_O/100 g dry solids), C was the model constant, and aw was the water activity. In the GAB model, Kg was the model constant. The monolayer content Wm was a measure of the availability of active sites for water sorption [19]. It represented the moisture content where dehydrated food materials showed their maximum shelf life since degradation reactions were limited [20]. Parameter C was related to the binding force of water to the primary binding sites. Regarding the Kg parameter, when this approached 1, there were practically no differences between the multilayer water molecules and those of free water [19].

### 2.9. Statistical Analysis

Statistical analysis was conducted using analysis of variance (ANOVA). Means were compared with an LSD Fisher test at a significance level of 0.05. INFOSTAT statistical software v2020 (*Facultad de Ciencias Agropecuarias*, UNC, Córdoba, Argentina) was utilized for the analysis.

## 3. Results and Discussion

### 3.1. Particle Size Distribution Analysis

Starches extracted from different plant sources display unique granular shapes and structures. Starch granules can vary in shape, including spherical, oval, polygonal, lenticular (disk-shaped), and elongated forms. Their sizes can range from less than 1 µm to 100 µm in diameter. Wheat starch displays a bimodal particle size distribution: the larger granules (A-type) are disk-shaped or lenticular, with diameters between 18 and 33 µm, while the smaller granules (B-type) are spherical, with diameters ranging from 2 to 5 µm [21].

**Figure 1:**
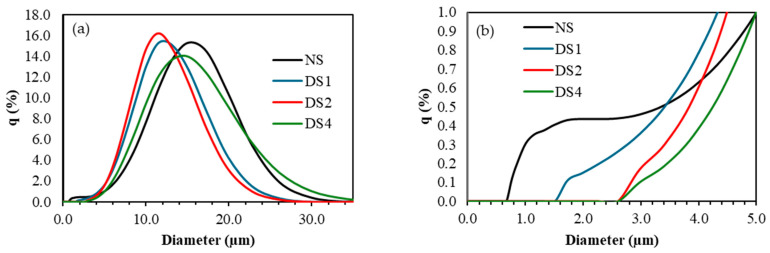
Particle size distributions of starch samples. (**a**) Complete particle size distributions, (**b**) B-type starch granules particle size distributions. q: volume (%). NS: 4.8% DS, DS1: 14.7% DS, DS2: 21.4% DS, DS4: 32.2% DS.

The particle size distribution of the starch granules is shown in Figure 1a. Increased mechanical damage to the starch granules caused a shift in the population of B-type granules toward larger particle diameters (Figure 1b). This shift can be attributed to a higher swelling in cold water or at ambient temperature. As a result, the peak corresponding to the B-type granule population began to overlap with that of the A-type granule population, making the bimodal distribution nearly indistinguishable. The rapid hydration and swelling of starch granules may occur due to alterations in their surface structure. The milling process can modify the granular surface, impacting the hydration and swelling of starch granules [8]. Previous studies have shown that the B-type granules in wheat starch are more susceptible to damage than A-type granules [22].

As for the A-type granule population, increased mechanical damage caused a shift toward smaller particle diameters, likely due to granular rupture. However, in the sample with the highest level of damage (DS4), the A-type granule population shifted toward larger diameters, approaching the distribution observed in the native starch sample. This suggests that A-type granules may become more prone to damage at higher grinding times, leading to their hydration, which was previously insignificant compared to B-type granules at lower grinding times. Hong et al. [23] reported that A-type wheat starch granules increase in size with prolonged regrinding (1, 3, and 5 h).

### 3.2. Fourier Transform Infrared Spectroscopy (FT-IR)

The FTIR spectra from 4000 to 700 cm^−1^ of the starch samples are shown in Figure S1. The spectrum region located between 4000 cm^−1^ and 1500 cm^−1^ showed the same characteristic peaks for all the analyzed samples. All four samples exhibited the same characteristic peaks at 3500 cm^−1^, 2900 cm^−1^, 2100 cm^−1^, and 1650 cm^−1^. The peak at 1650 cm^−1^ was associated with the water molecules absorbed in the amorphous region [24]. The peak at 2100 cm^−1^ is attributed to free water content [25]. The peaks at 2900 and 3500 cm^−1^ correspond to CH_2_ deformation and OH bonds, respectively [26].

**Figure 2:**
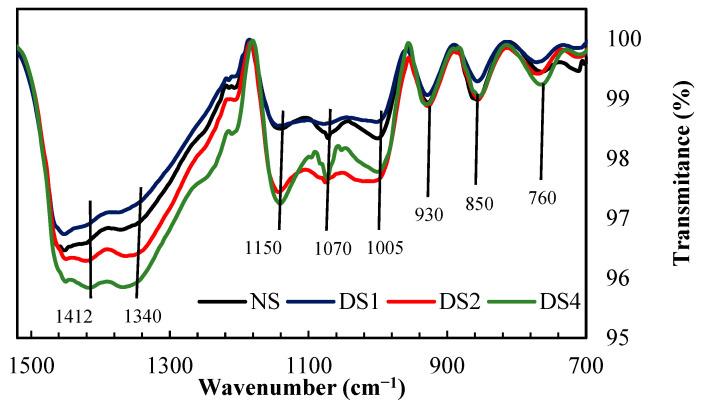
FT-IR spectrum “fingerprint” region of starch samples. q: volume (%). NS: 4.8% DS; DS1: 14.7% DS; DS2: 21.4% DS; DS4: 32.2% DS.

The most important structural differences could be observed in the region located between 1500 and 700 cm^−1^ (Figure 2), considered the “fingerprint”. These are related to information about changes in the polymer structure and starch conformation. The peaks at approximately 1412 cm^−1^ were assigned to the bending vibrations of the -CH_2_ bonds and the stretching of the O-C-O bonds. The bands observed near 1340 cm^−1^ correspond to the bending vibrations of the C-O-H bonds and the twisting vibrations of the -CH_2_ bond. Around 1150 cm^−1^, a band was observed to be assigned to the vibrations of the C-O-C glycosidic bonds and the complete glucose ring that may be present in different vibrational modes and bending conformations [27]. At 1070 cm^−1^ and 1005 cm^−1^, the spectra presented two bands assigned to the crystalline and amorphous regions of starch, respectively. The peak observed at 930 cm^−1^ was assigned to the vibrational modes of the α-(1→4) glycoside bonds of the starch glucose chain backbone. Finally, the signals observed at 850 cm^−1^ and 760 cm^−1^ correspond to the deformation of the C bonds (C1 of the glucose structure)-H and CH_2_, and the stretching vibrations of the C-C bonds [27].

Some authors have suggested that the infrared spectroscopy technique is sensitive to the so-called “short-range order”, defined as the order of the double helix of the starch structure, as opposed to the “long-range order” related to the packing of the double helices [28].

An increase in the intensity of the band located at around 1005 cm^−1^ has been associated with a decrease in the crystallinity of starch. On the other hand, an increase in the intensity of the band located at around 1070 cm^−1^ has been associated with an increase in the crystallinity of starch. The ratio (R) between the intensities (I) of the bands (R = I1070/I1005) has been analyzed to study changes in the crystallinity of starch granules because of different processes [26,29]. In this work, a decrease in the intensity of the band to 1070 cm^−1^ and an increase in the intensity of the band to 1005 cm^−1^ were observed, and therefore a decrease in the R ratio with the increase in the content of damaged starch. This result suggests that, by increasing the level of mechanical damage of the granules, the amorphous proportion of the polymeric structures that make up the granular arrangement increases.

### 3.3. X-Ray Diffraction (XRD)

Radial confirmation of amylopectin is the basis of the structure of starch granules [2]. Taking this into account, the short chains of amylopectin are what largely determine the level of crystallinity of the starch granules.

**Figure 3:**
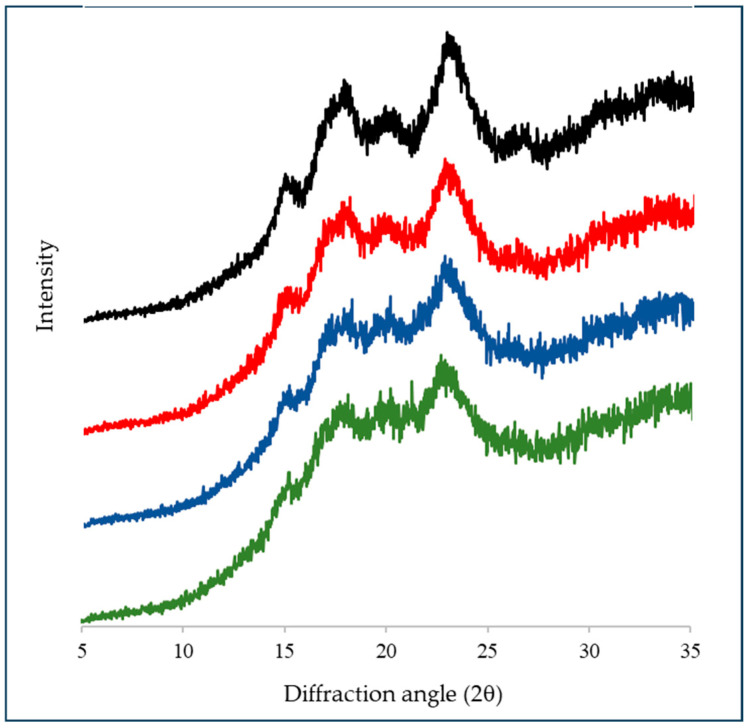
X-ray diffraction patterns of starch samples. NS: 4.8% DS (black); DS1: 14.7% DS (red); DS2: 21.4% DS (blue); DS4: 32.2% DS (green).

The diffraction patterns of starch samples with different damaged starch content showed the characteristic profiles of starch from cereals (A-type) (Figure 3). The main refraction peaks of this type of pattern are observed at 2°, 15° and 23°, and a doublet at approximately 17° and 18° [30]. The type of crystalline polymorph is determined by how the double helices of the amylopectin chains are organized and packed in the starch granule and by the average length of amylopectin chains. Wheat starch exhibits this diffraction pattern because, on average, it has short chain lengths [31].

The intensity of the peaks in the diffractograms decreased with the amount of damaged starch (Figure 3). These findings suggest a loss of structural order in the starch granules. In agreement with these results, it has been shown that starch crystallinity, measured by X-ray diffraction, differential scanning calorimetry, and nuclear magnetic resonance, depends on the severity of the milling conditions and time [32].

The degree of total crystallinity of the starch was determined from the deconvolution of the peaks obtained from the diffraction patterns, and then calculating the integrals of the areas of the amorphous and crystalline peaks. The degree of total crystallinity for the starch samples with different damaged starch content gradually decreased (*p* < 0.05) from 34.1% (NS) to 32.3%; and 30.4% and 28.9% for the DS1, DS2, and DS4 samples, respectively. These results confirm that there was a loss of crystallinity of the damaged starch granules due to mechanical action. Franzoni et al. [33] found similar crystallinity values for starch samples with different contents of damaged starch, using low-field and single-sided NMR analysis.

Morrison et al. [34] observed a similar behavior and proposed that mechanical damage to starch granules causes a gradual loss of the molecular order of the polymers that form it. These authors noted that the crystallinity determined by X-ray diffraction and the double helix content of amylopectin decrease as the damaged starch content increases, leading to the idea that mechanical damage could produce a new amorphous material, which would be differentiated from the amorphous part of the native starch granules. The gelatinization enthalpy values correlated with the decrease in the crystallinity of the starch granules, as observed in previous studies [3,35]. The enthalpy of gelatinization of starch granules involves different thermodynamic events that include the swelling of the granules, fusion of crystals (endothermic processes), and hydration and rearrangement of the polymer chains (exothermic processes) [32].

Various authors have reported that mechanically treating starch samples results in defects accumulating in the crystal structure. Dome et al. [36] stated that the grinding process disrupts the double helices of amylopectin and the single helices of amylose. This disruption occurs due to the breaking of hydrogen bonds or glycosidic bonds within the crystalline regions. Therefore, milling causes the release of intercalated amylose molecules from the crystalline regions into the amorphous regions [36].

### 3.4. Scanning Electron Microscopy (SEM)

**Figure 4:**
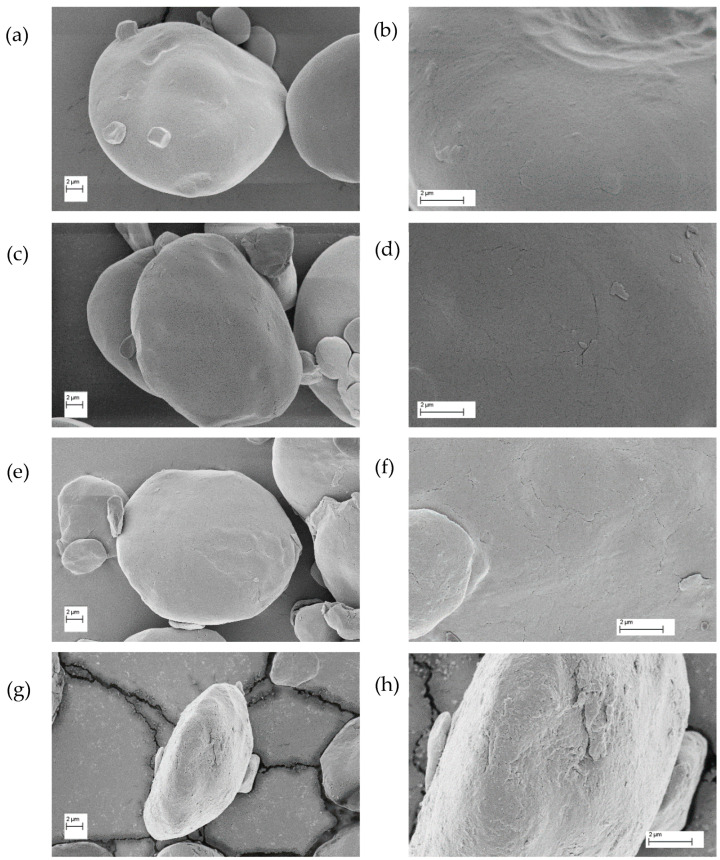
SEM images of starch samples. On the left: 3.00 K X magnification. On the right: 8.00 K X magnification. (**a**,**b**) NS: 4.8% DS, (**c**,**d**) DS1: 14.7% DS, (**e**,**f**) DS2: 21.4% DS, (**g**,**h**) DS4: 32.2% DS. (**a**,**c**,**e**,**g**) images: 3 kV, 3000×. (**b**,**d**,**f**,**h**) images: 3 kV, 8000×.

Scanning electron microscopy (SEM) is a widely used tool to study the structural characteristics and architecture of starch granules. Although image acquisition requires a high vacuum and sample preparation techniques that tend to damage or alter the structure of the granules [2], this microscopic technique has proven to be very useful for studying the surface and the internal structure of starch granules. A disadvantage of the SEM technique is that the morphological analysis obtained from the images is only qualitative. The images obtained using the SEM showed the presence of large and lenticular and small and spherical starch granules, which correspond to A-type and B-type granules, respectively (Figure 4).

The analysis of the images of the native starch (NS) sample revealed that the surface of the granules was smooth and flat, although there were reliefs or marks associated with points where the granules could have been in close contact with another one [8,22]. However, in the images of samples DS1, DS2, and DS4, it was observed that the surface of the granules went from having a smooth and polished appearance to a rough and flaky appearance, because of the grinding process (Figure 4). Similarly, Tester et al. [22] reported that, as a consequence of grinding, the granular surfaces became distorted, rough, and with numerous clumps, and in this sense, few intact granules after this process were observed.

### 3.5. Water Absorption Capacity (WAC)

Damage to starch granules during milling increased their water absorption capacity (Table 1). However, the swelling power decreases when damaged starch granules are generated under extremely severe conditions [37]. Since milling can promote an alteration in the proportion of hydrogen bonding interactions, it is possible that, because of this phenomenon, more hydroxyl groups within the double helices become available to interact with the surrounding water molecules [38], thus increasing the water absorption capacity (and, at the same time, increasing its swelling power). Likewise, damaged starch granules have a structurally altered surface, which facilitates the access of water molecules, which consequently promotes their absorption [39]. However, excessive granular damage during milling may cause the collapse of the semi-crystalline layers and contribute to the decrease in the swelling power of the granules.

Wang and Copeland [40] suggested that the integrity of amylopectin dominates the swelling power of starch granules. Presumably, milling depolymerized amylose chains, which could result in a decrease in the swelling power of the granules, with the increase in the degree of milling [39]. From this, it is possible to infer that damaged starch may have higher solubility compared to native starch. The greater the severity of milling (and, consequently, the higher the damaged starch content), the greater the starch solubility [37,41]. Some authors have observed that solubility is positively correlated with damaged starch content [42]. The depolymerization of starch granules caused by the mechanical milling process increases the proportion of molecules of lower molecular weight and consequently increases their solubility. González et al. [43] observed a negative exponential correlation between the solubility and the degree of crystallinity of rice starch. According to this, the structural disorder (associated with the increase in the amorphous regions of the starch granules) caused by the reductions in the crystalline regions also contributed to the increase in solubility. Furthermore, the solubility of damaged starch increases noticeably in hot water (T° > 50 °C) in contrast to water at room temperature (~25 °C) [44]. The reason for this behavior could be that the molecules move faster at higher temperatures, which accelerates the dispersion of amylose from the amorphous region and other water-soluble factions.

### 3.6. Thermogravimetric Analysis (TGA)

**Figure 5:**
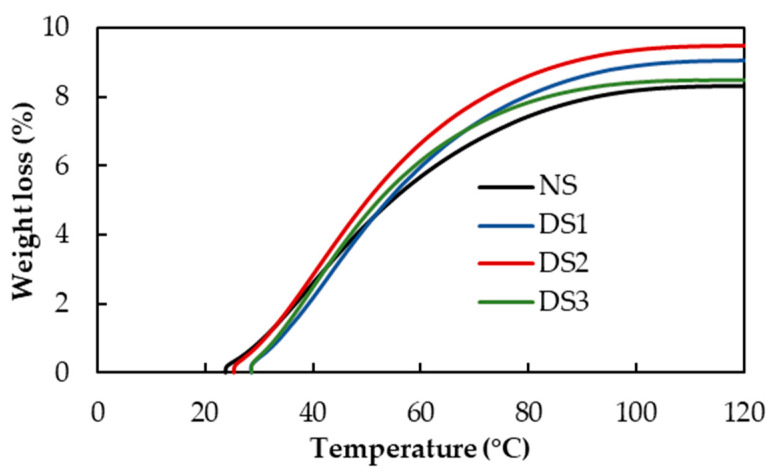
Curves of the percentage of weight loss as a function of temperature for starch samples, obtained by TGA. NS: 4.8% DS; DS1: 14.7% DS; DS2: 21.4% DS; DS4: 32.2% DS.

Thermogravimetric analysis (TGA) is a thermal analysis method based on the measurement of the mass loss of a material as a function of temperature. The observed changes in thermal behavior can be attributed specifically to the evaporation of water from the system, due to heating. The curves of the percentage of weight loss (%) as a function of the temperature of the samples are shown in Figure 5. The percentage of weight loss at different temperatures is shown in Table 2.

According to the results, the percentage of water lost at 50 °C and 70 °C increased gradually and significantly (*p* < 0.05) with the increase in the damaged starch content. These results suggest that, as damage to the starch granule structures increased, the water bound to them was released at a higher rate. In addition, the temperature at which different percentages of water were lost (30, 50, 75, 80, 90, and 100%) was determined, which are detailed in Table 3. As a general trend, it can be observed that the temperature at which a certain percentage of water was lost from the samples decreased with the increase in the damaged starch content. This effect was more significant at water percentages of 75, 80, and 90%. As in the analysis previously carried out, the results indicated that, by increasing the content of damaged starch, water was released faster, that is, at lower temperatures.

**Figure 6:**
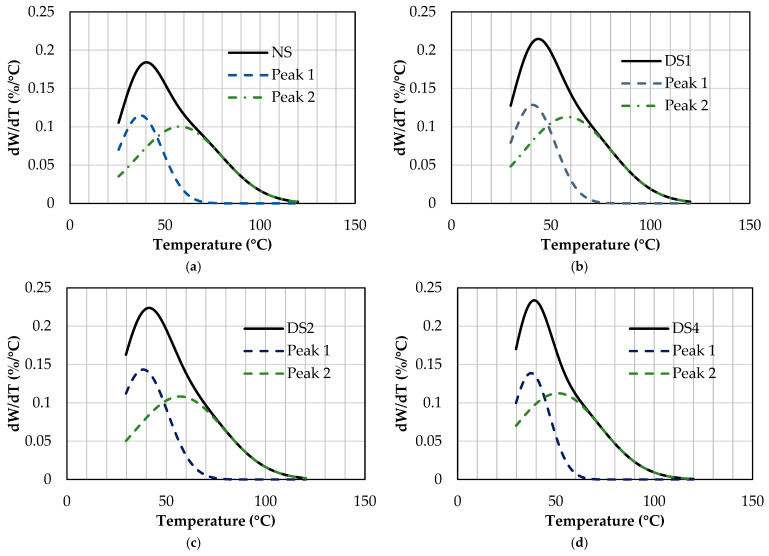
Curves of derivate of weight loss as a function of temperature (dW/dT) for starch samples, obtained by TGA. (**a**) NS: 4.8% DS; (**b**) DS1: 14.7% DS; (**c**) DS2: 21.4% DS; (**d**) DS4: 32.2% DS.

The DTGAs curves (Figure 6) seem to reflect the presence of two overlapping events, a main peak at around 45 °C and a second one (a small shoulder of the first one) at around 60 °C. Two peaks were obtained for each starch sample, indicating the existence of two populations of water: one population that presents weaker interactions with the starch granules (population 1) and another population that shows stronger interactions with them (population 2). Table 4 shows the temperatures at which the main peak occurs for each sample and the areas of each peak relative to the total area of the curves. Water in the presence of heat establishes a high-pressure vapor gradient within the granular structure. Starch retains water through the hydrogen bonds between amylose and amylopectin branches and inter-amylopectin helices [45].

After the elimination of excess water, drying speeds always follow three well-defined stages: the first stage is related to the evaporation of interstitial water between the granules, which evaporates almost as pure water; the second stage is associated with the surface water of the granules; and finally, the third stage relates to the most strongly bound water (or structural water). The water molecules most tightly bound to starch can be interpreted as water in the crystalline regions and the amorphous regions of the starch granules.

Water population 1 presented maximum peak temperatures between 41.7 °C (NS) and 37.7 °C (DS4), with a significant decrease observed as the damaged starch content increased. On the other hand, water population 2 also showed a gradual and significant (*p* < 0.05) decrease in peak maximum temperatures from 61.5 °C (NS) to 51.7 °C (DS4). Because water population 1 appeared at lower temperatures, it can be related to excess water, the interstitial (located between the granules), and the water found on the surface of the starch granules. The water population 2 can be attributed to those molecules that are more closely bound to the starch in the pores and channels of the granules, associated with the amorphous and crystalline regions.

By increasing the damaged starch content from 4.8 to 32.2%, a decrease in the peak temperature of both populations was detected. However, this decrease was greater for the peak of water population 2 (10.2 °C for population 2 and 3 °C for population 1). In contrast, the increase in damaged starch content caused a gradual and significant increase (*p* < 0.05) in the relative area of population 1, and a gradual and significant (*p* < 0.05) decrease in the relative area of population 2. The area under the curve gives us information about the number of water molecules associated with that population.

In summary, the increase in the content of damaged starch caused different effects: (a) an increase in the proportion of less bound water and a decrease in its evaporation temperature (fraction of water found on the surface of the granules and in the interstitial regions) and (b) a decrease in the amount of more strongly bound and in its evaporation temperature (fraction of water found in the crystalline and amorphous regions of the granules). Consequently, the greater the degree of damage to the starch granules, the more easily water was released, and the proportion of less bound water increased.

### 3.7. Dynamic Vapor Sorption (DVS)

The relationship between the total moisture content and the corresponding water activity of a product at a constant temperature generates what is called a sorption isotherm [46].

**Figure 7:**
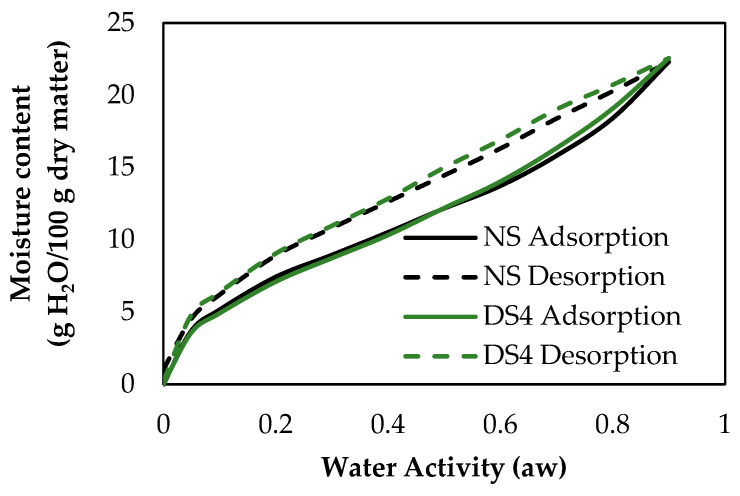
Sorption isotherms for NS and DS4 starch samples. NS: 4.8% DS; DS4: 32.2% DS.

All the curves showed an increase in the equilibrium moisture content with increasing water activity (aw) (Figure 7 shows those obtained for NS and DS4). The sigmoidal shape found in all isotherms of the samples is typical of type II isotherms based on Brunauer’s classification [47]. This type of isotherm is due to the monolayer–multilayer sorption theory.

The DVS curves obtained for the samples with different damaged starch content were similar, although, a change in the increasing slope of the curve was observed from the aw values of 0.7. This upward curvature can result from the condensation of vapor molecules within the interstitial spaces of materials or polymers. The sorption of water by starch occurs through the interaction of water molecules with hydroxyl groups, which are primarily found in the amorphous regions and on the surface of starch crystals. In contrast, the crystalline regions of starch typically resist moisture penetration and have energetically less favorable sites for water sorption [48]. However, in the aw region above 0.7, the sorption patterns showed different slopes of increasing moisture content with increasing water activity. Comparing the adsorption process of the NS and DS4 samples, above an aw of 0.7, a higher water adsorption was observed when the damaged starch content was 32.2% (DS4) (Figure 7). This was inversely related to the crystallinity percentages of the starch samples, since the DS4 sample presented a lower crystallinity (determined by XRD), and therefore, an increase in the proportion of amorphous regions.

In the wet state prior to desorption, nearly all the hydroxyl groups in the molecular structure of the starches were occupied by absorbed water molecules. During the desorption process, these hydroxyl groups were gradually released, allowing them to come closer together and form hydrogen bonds with each other. Through this interaction, the interstitial spaces of the polymers that formed starch were irreversibly reduced, so that the trapping of capillary water molecules decreased. Some researchers have suggested that irreversible polymer shrinkage may create kinetic barriers to moisture migration, which could account for the observed “non-zero” moisture contents at aw = 0 during desorption [48].

**Figure 8:**
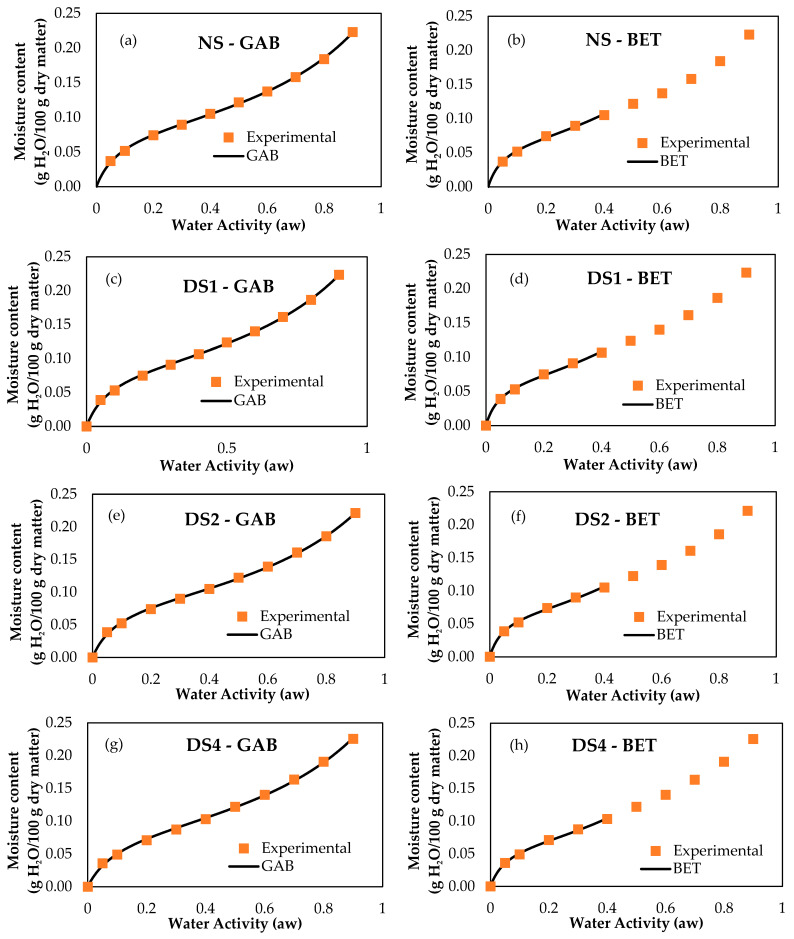
The sorption curve fits with the GAB and BET models for the starch samples. (**a**,**b**) NS: 4.8% DS; (**c**,**d**) DS1: 14.7% DS; (**e**,**f**) DS2: 21.4% DS; (**g**,**h**) DS4: 32.2% DS.

The two analyzed models fitted the experimental data (correlation coefficients > 0.99) related to the water adsorption process. Figure 8 shows the curves obtained experimentally and those obtained through mathematical adjustments for the GAB and BET models. The adjustment parameters of the isotherms from the models applied for water adsorption are shown in Table 5. The monolayer value (associated with the parameter Wm) for the damaged starch samples was estimated using the GAB and BET models. The monolayer values were similar for all the starch samples, ranging from 0.086 to 0.090 g water/g solids and 0.068 to 0.070 g water/g solids for GAB and BET, respectively. However, a decrease in the value of parameter C was observed when DS increased from 4.8 to 32.2%. The C value is related to temperature and isosteric heat (also known as differential enthalpy of sorption, which provides a measure of the energy of the union between the adsorbed water and the molecules of food) during the adsorption of water in food. In the GAB model, this decrease was observed gradually, from 18.1 for NS to 14.5 for DS4. While in the BET model, the decrease was from 20.1 to 18.6 for AN and DS4, respectively. These modifications suggested that the driving force for the monolayer attachment also decreased. Therefore, the energy involved in the unions of the starch polymer chains with the adsorbed water was lower with increasing mechanical damage.

## 4. Conclusions

The damage caused by the milling process of the starch granules produced physical changes in their structures. These modifications could be observed qualitatively by scanning electron microscopy, where more irregular, rough, and less uniform granule surfaces were distinguished compared to those of the native starch granules. Analyzing the particle size distribution, it was concluded that the B-type and A-type granules were susceptible to the effects of the mechanical damage produced during the milling times analyzed.

Different techniques used during this stage of the work (X-ray diffraction and infrared spectroscopy) allowed us to confirm that there was a decrease in the crystallinity of the starch granules by the milling process. This loss of the degree of crystallinity led to modifications in the interactions with water molecules.

TGA and WAC analyses suggested that the increase in the content of damaged starch generated a greater absorption of water and that the interactions established between water and the polymers of the damaged starch samples were weaker than those established with native starch. Consequently, the greater the degree of damaged starch, the higher the proportion of the less bound water and the more easily water was released. Additionally, DVS analysis showed a higher absorption of water at high aw values and a lower energy involved in the unions of the starch polymer chains with the adsorbed water when increased the mechanical damage.

The main findings of this research provide a deeper understanding of the changes associated with the mechanical damage of the starch granular structure and its interaction with water. Further studies are needed to relate these results to the hydration properties of flours and their relationship with the rheological and thermal characteristics of starch-based systems.

## Figures and Tables

**Table 1:** Effect of DS on water absorption capacity of starch.

Sample	WAC
NS	1.93 ± 0.01 ^a^
DS1	2.28 ± 0.05 ^b^
DS2	2.53 ± 0.01 ^c^
DS4	2.76 ± 0.04 ^d^

WAC: water absorption capacity. NS: 4.8% DS; DS1: 14.7% DS; DS2: 21.4% DS; DS4: 32.2% DS. Values followed by different letters are significantly different (*p* < 0.05).

**Table 2:** Effect of DS on weight loss at 30, 50, 70, 90, 100, and 110 °C measured by TGA.

Sample	Weight Loss (%)					
	30 °C	50 °C	70 °C	90 °C	100 °C	150 °C
NS	4.1 ± 0.1 ^a^	43.1 ± 0.5 ^a^	77.1 ± 1.0 ^a^	94.3 ± 1.0 ^a^	97.8 ± 0.3 ^a^	99.7 ± 0.2 ^a^
DS1	4.8 ± 0.2 ^ab^	47.7 ± 0.9 ^b^	79.7 ± 0.5 ^b^	94.7 ± 0.1 ^ab^	98.0 ± 0.1 ^ab^	99.6 ± 0.0 ^a^
DS2	9.0 ± 0.6 ^c^	53.3 ± 1.3 ^c^	82.6 ± 0.6 ^c^	95.7 ± 0.3 ^bc^	98.5 ± 0.2 ^bc^	99.6 ± 0.1 ^a^
DS4	5.8 ± 0.6 ^b^	55.5 ± 1.9 ^c^	84.7 ± 0.4 ^d^	96.7 ± 0.1 ^c^	98.9 ± 0.2 ^c^	99.5 ± 0.1 ^a^

NS: 4.8% DS; DS1: 14.7% DS; DS2: 21.4% DS; DS4: 32.2% DS. Values followed by different letters in the same column are significantly different (*p* < 0.05).

**Table 3:** Effect of DS on temperature at 30, 50, 75, 80, 90, and 100% of weight loss measured by TGA.

Sample	Temperature (°C)					
	30%	50%	75%	80%	90%	100%
NS	40.1 ± 0.9 ^a^	51.2 ± 2.9 ^a^	67.1 ± 2.5 ^b^	71.5 ± 2.4 ^c^	82.8 ± 2.1 ^c^	165.3 ± 43.9 ^a^
DS1	42.3 ± 0.4 ^a^	51.1 ± 0.5 ^a^	66.2 ± 0.4 ^b^	70.3 ± 0.4 ^bc^	81.8 ± 0.3 ^bc^	195.8 ± 0.3 ^a^
DS2	39.7 ± 0.6 ^a^	48.5 ± 0.6 ^a^	63.3 ± 0.7 ^ab^	67.5 ± 0.6 ^ab^	78.9 ± 0.7 ^ab^	196.7 ± 0.8 ^a^
DS4	39.8 ± 0.8 ^a^	47.5 ± 1.0 ^a^	61.5 ± 0.7 ^a^	65.6 ± 0.5 ^a^	76.5 ± 0.3 ^a^	192.9 ± 5.2 ^a^

NS: 4.8% DS; DS1: 14.7% DS; DS2: 21.4% DS; DS4: 32.2% DS. Values followed by different letters in the same column are significantly different (*p* < 0.05).

**Table 4:** Effect of DS on peak temperature (T) and relative peak area percentages (A) for the deconvolved peaks measured by TGA.

Sample	T1 (°C)	A1 (%)	T2 (°C)	A2 (%)
NS	41.7 ± 0.5 ^b^	37.5 ± 0.3 ^a^	61.5 ± 0.3 ^c^	62.5 ± 0.3 ^c^
DS1	40.4 ± 0.4 ^b^	38.7 ± 0.5 ^b^	58.2 ± 0.3 ^b^	61.3 ± 0.5 ^b^
DS2	37.9 ± 0.7 ^a^	42.5 ± 0.2 ^c^	56.4 ± 0.8 ^b^	57.5 ± 0.2 ^a^
DS4	37.7 ± 0.5 ^a^	43.0 ± 0.3 ^c^	51.7 ± 0.9 ^a^	57.0 ± 0.3 ^a^

T1 and A1 correspond to peak 1. T2 and A2 correspond to the peak. NS: 4.8% DS; DS1: 14.7% DS; DS2: 21.4% DS; DS4: 32.2% DS. Values followed by different letters in the same column are significantly different (*p* < 0.05).

**Table 5:** Effect of DS on parameters determined from the fit of the GAB and BET models to the experimental data for starches measured by DVS.

Model	Sample	W_m_	K_G_	C	R^2^	s
GAB	NS	0.086	0.693	18.1	0.99	0.0013
	DS1	0.089	0.685	18.4	0.99	0.0015
	DS2	0.088	0.684	18.0	0.99	0.0016
	DS4	0.090	0.690	14.5	0.99	0.0022
BET	NS	0.069		20.1	0.99	0.0017
	DS1	0.070		21.5	0.99	0.0016
	DS2	0.068		21.3	0.99	0.0016
	DS4	0.068		18.6	0.99	0.0014

W_m_: monolayer moisture content; Kg and C: model constants; s is the standard deviation of the fit. NS: 4.8% DS; DS1: 14.7% DS; DS2: 21.4% DS; DS4: 32.2% DS.

## Data Availability

The original contributions presented in this study are included in the article/Supplementary Materials. Further inquiries can be directed to the corresponding author.

## Supplementary Materials

The following supporting information can be downloaded at https://www.mdpi.com/article/10.3390/foods14010021/s1, Figure S1. FT-IR spectrum of starch samples.
