# Supplementary figures and images for: The Properties of Damaged Starch Granules: The Relationship Between Granule Structure and Water–Starch Polymer Interactions
Source: Foods. 2024 Dec 25;14(1):21. doi: 10.3390/foods14010021 (PMC11719984; doi:10.3390/foods14010021)

## Supplementary Materials

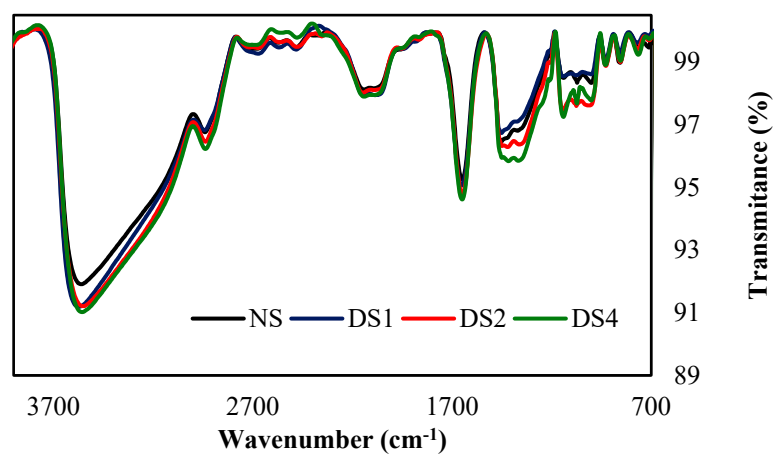

**Figure S1.** FT-IR spectrum of starch samples

Supplement: Supplementary file 1 [file foods-14-00021-s001.zip › foods-3366080-supplementary.pdf]
